# Toxic Erythema of Chemotherapy, Vasculitic Eruption With Malignant Intertrigo Characteristics, and Superimposed Infection Post-bevacizumab Initiation

**DOI:** 10.7759/cureus.52816

**Published:** 2024-01-23

**Authors:** Kristin N Slater, Trevor Nessel, Francisca Kartono

**Affiliations:** 1 Dermatology, Lincoln Memorial University DeBusk College of Osteopathic Medicine, Harrogate, USA; 2 Dermatology, Corewell Health, Farmington Hills, USA; 3 Dermatology, MI Skin Center Dermatology Clinic, Northville, USA

**Keywords:** nsclc, alk+ non-small cell lung carcinoma, lorlatinib, bevacizumab, adverse drug reaction, drug reaction, vasculitis, malignant intertrigo, tec, toxic erythema of chemotherapy

## Abstract

Drug reactions are a known risk in combined anti-cancer therapy. Less commonly recognized risks of chemotherapies and targeted immunotherapies include toxic erythema of chemotherapy reactions. With the immunosuppressive quality of cancer combined with anti-cancer treatments, patients are also susceptible to increased infection. We report a rare case of combined targeted anti-cancer treatment with bevacizumab and lorlatinib, and an associated transformation of an eczematous process into a toxic erythema of chemotherapy vasculitic eruption, with combined malignant intertrigo characteristics and superimposed infection following the initiation of bevacizumab.

## Introduction

Bevacizumab, a monoclonal antibody, is a targeted molecular anti-cancer treatment that inhibits tumor angiogenesis by blocking vascular endothelial growth factor (VEGF) [1]. Because of its effect on angiogenesis, bevacizumab has been reported in isolated cases to cause vascular-related side effects, including thrombotic microangiopathy-like lesions secondary to endothelial damage in the glomerulus [1], one published case documenting bevacizumab-induced IgA vasculitis and nephritis [2], and a case of drug-induced aortitis potentially caused by bevacizumab [3]. These cases are rare but notable. Other noteworthy cutaneous reactions related to bevacizumab include exfoliative dermatitis, an erythematous papular rash (noted with intravitreous use), and ulceration of striae [4]. Bevacizumab can be used in conjunction with other agents, such as alectinib or other tyrosine kinase inhibitors [5]. Lorlatinib is an anaplastic lymphoma kinase (ALK) inhibitor used in advanced-stage ALK+ non-small cell lung carcinoma (NSCLC) [6]. Lorlatinib and Bevacizumab can be combined to treat metastatic ALK+ NSCLC, but there is limited data regarding the use and side effect profile of this treatment combination [7]. Cases of toxic erythema of chemotherapy (TEC) are rarely reported, often misdiagnosed, and consequentially result in hospitalizations [8]. While TEC reactions have not, to our knowledge, been reported related to bevacizumab alone, they have been reported with bevacizumab in combination with leucovorin, 5-fluorouracil, and irinotecan (FOLFIRI) [8]. With this combination therapy, a TEC reaction in a malignant intertrigo pattern was seen [8]. TEC reactions have also been reported with the drug axitinib alone [8]. Axitinib is a multikinase inhibitor, with a related inhibition target to bevacizumab, a vascular endothelial growth factor receptor [9]. In axitinib, there have been reports of malignant intertrigo, a subset of TEC, which involves the intertriginous areas, and other cutaneous reactions [8-9]. We report a case of a vasculitic eruption suspected to be a TEC reaction, with malignant intertrigo characteristics and superimposed infection following the addition of bevacizumab to lorlatinib in a patient with stage 4 ALK+ NSCLC.

## Case presentation

**Figure 1:**
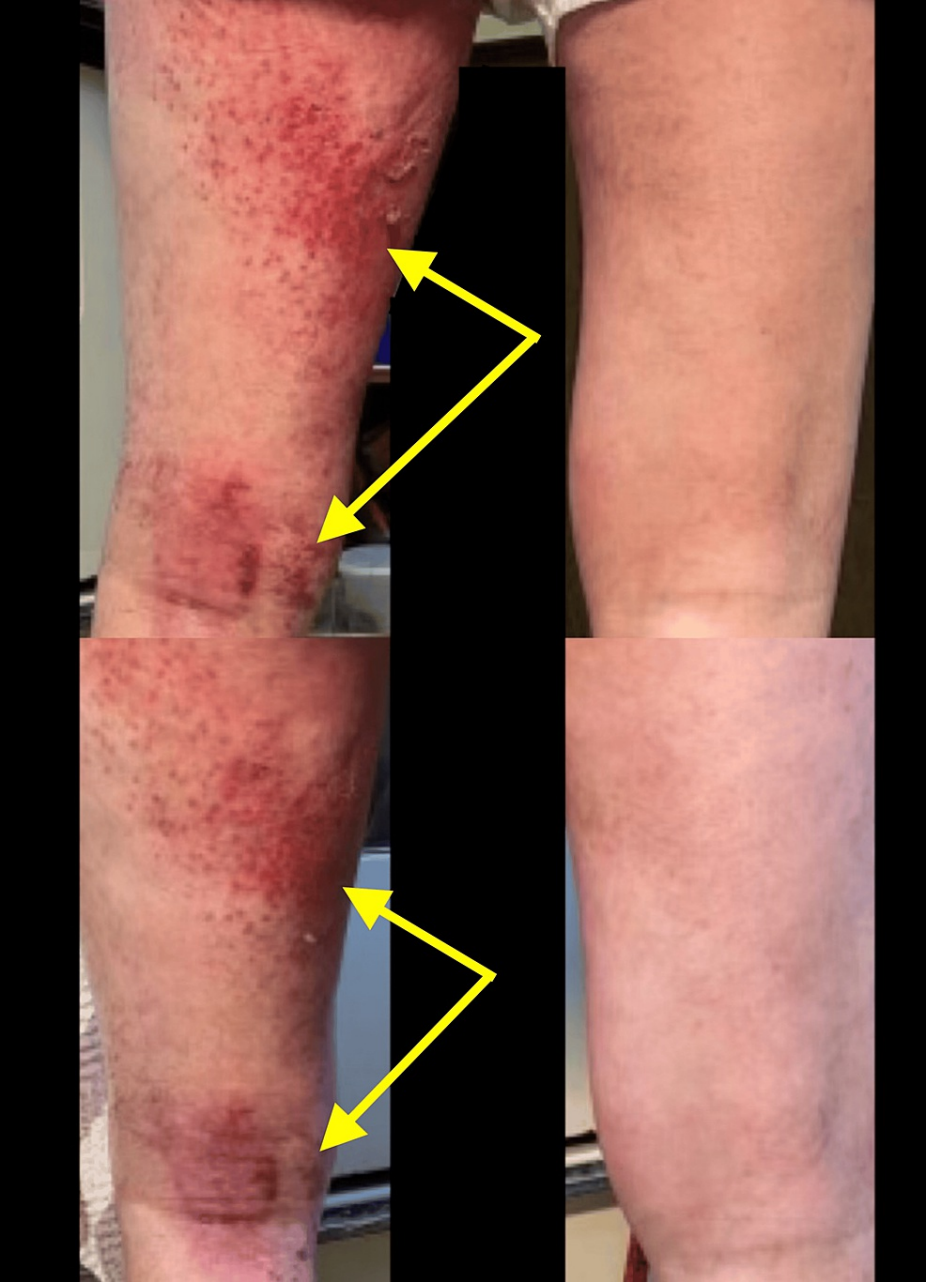
Two photos side by side showing the eczematous itchy grouped scaly erythematous macules and patches predominantly on the left posterior medial thigh and left popliteal fossa, compared with the right posterior leg

**Figure 2:**
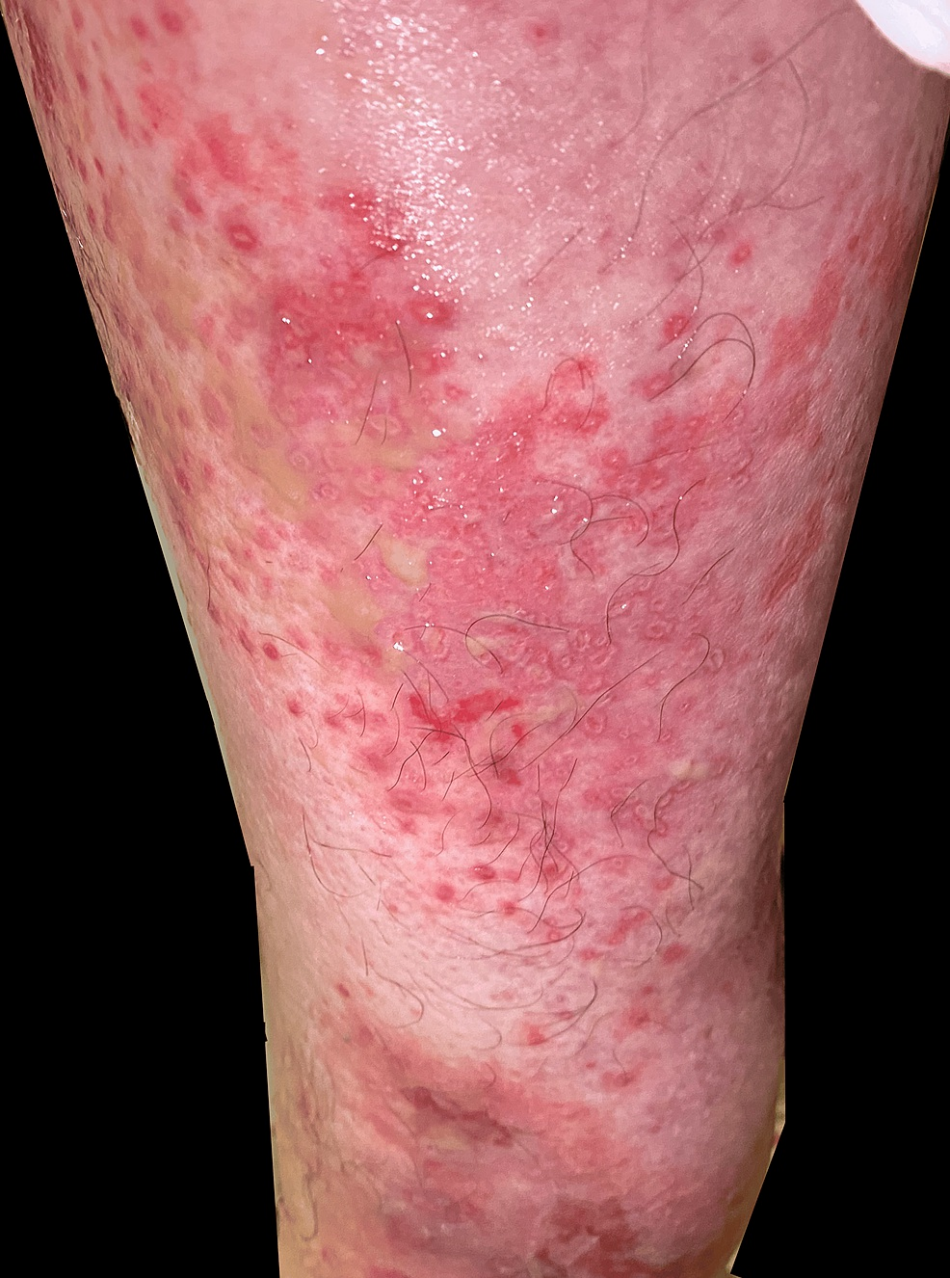
Grouped draining pustules superficial erosions on the left posterior medial thigh and left knee L1-L3/zosteriform look and distribution

**Figure 3:**
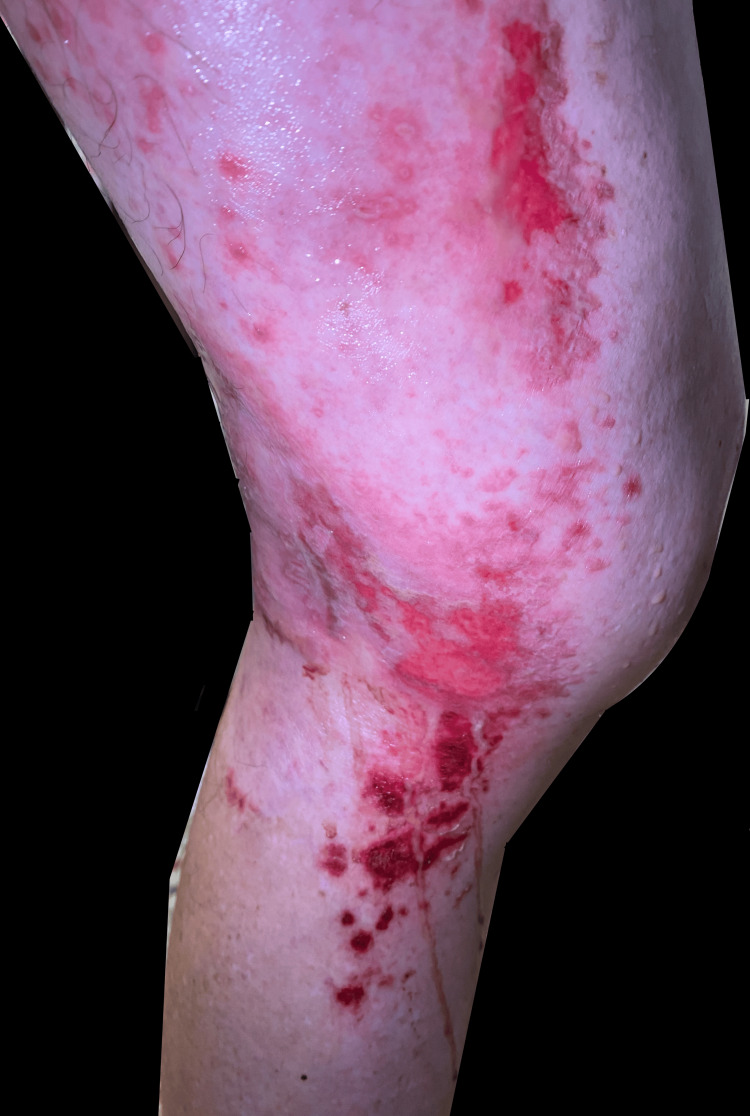
Grouped draining pustules superficial erosions on the left posterior medial thigh and left knee L1-L3/zosteriform look and distribution

**Figure 4:**
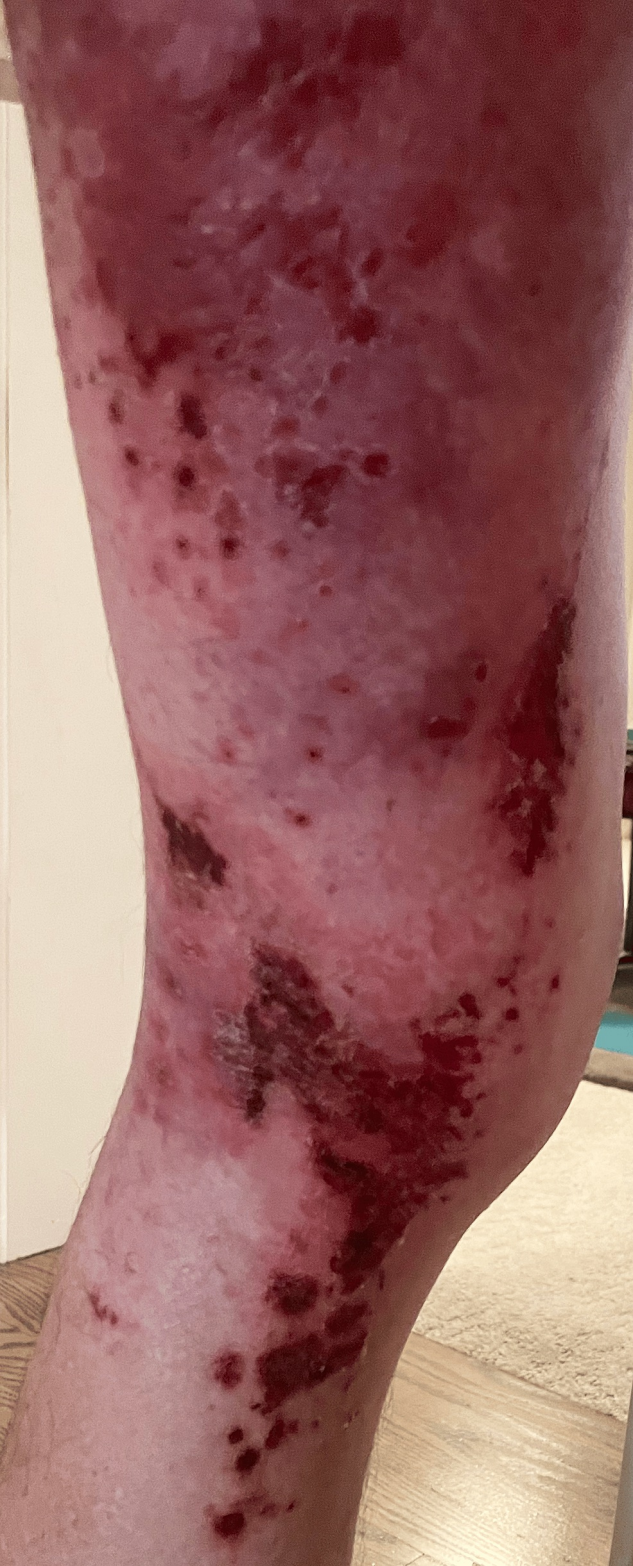
Medial view left leg: initial improvement after acyclovir, erythematous plaques with superficial erosions in the same distribution (on the left posterior medial thigh and left knee L1-L3 distribution)

**Figure 5:**
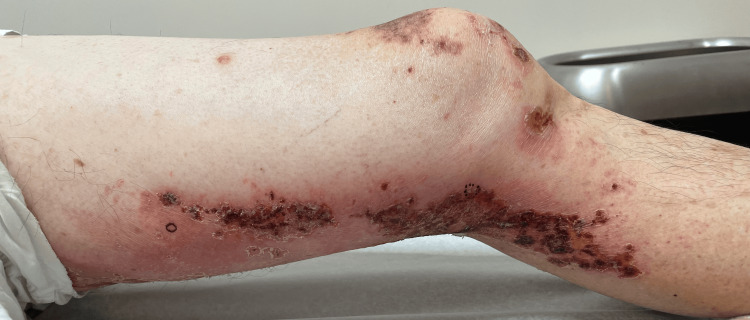
Left medial leg with erythema surrounding the erosive plaques in the same distribution seen in the days prior. This photograph was taken the day of the biopsy and two days after the patient worsened and was switched to valaciclovir

**Figure 6:**
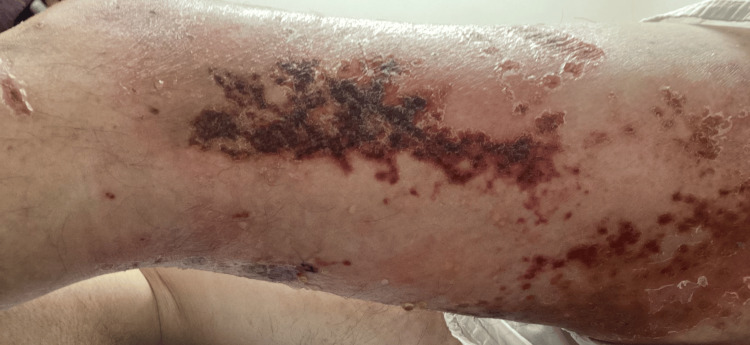
Close-up image of the left lateral leg with erythema surrounding the erosive plaques in the same distribution seen in the days prior. The photograph was taken the day of the biopsy and two days after the patient worsened and was switched to valaciclovir

**Figure 7:**
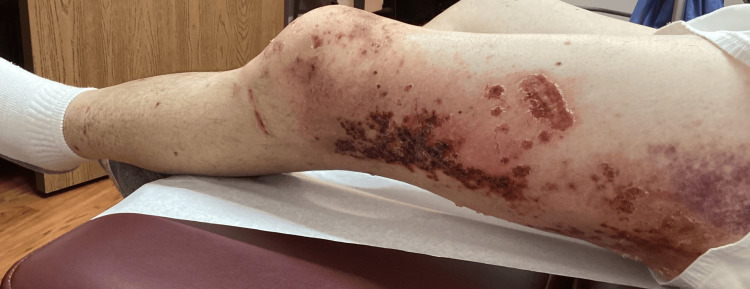
Left lateral leg with erythema surrounding the erosive plaques in the same distribution seen in the days prior. The photograph was taken the day of the biopsy and two days after the patient worsened and was switched to valaciclovir

A 70-year-old male with stage 4 NSCLC, adenocarcinoma with ALK+ mutation, presented with bilateral eczematous dryness of the legs. He maintained his NSCLC for two years using alectinib 600mg twice daily but began feeling unwell and had an MRI of the brain showing lesions. He was treated with cyberknife radiation and switched to lorlatinib at that time. Six months after beginning lorlatinib he presented with bilateral eczematous changes in the legs (Figure 1), which he treated with topical steroids and saw resolution of the right leg and left leg improvement. The following day the patient began his first bevacizumab infusion, with trimethoprim-sulfamethoxazole prophylaxis. Nine days after the initiation of bevacizumab the patient began experiencing a transformation of the residual eczematous rash into weeping wet ulcers, some targetoid lesions on the left anterior thigh, anterior knee, left medial thigh, and crusted papules on the ankles medially and laterally (Figures 2-3). Serous drainage was noted on the left thigh and mild erythema on the right thigh. The patient was started on oral acyclovir 400mg three times daily for probable eczema herpeticum, with 50% improvement (Figure 4); however, new areas of erythema including the left scrotum and left medial thigh were appreciated and the patient was switched to valaciclovir 1g three time daily (Figures 5-7).

A punch biopsy was taken from the left anterior distal thigh and the left medial proximal thigh at this time. The dermatopathology results from the left anterior distal thigh showed a punch of skin with prominently ulcerated epidermis surfaced by inflammatory scale crust. Where the epidermis was intact, there was superficial spinous layer necrosis which was ultimately surfaced by basket woven orthokeratin; squamatization of the basal layer with occasional necrotic keratinocytes was noted. The dermis contained sparse superficial perivascular infiltrates of lymphocytes, histiocytes, and occasional neutrophils, along with extravasated erythrocytes. There was fibrinoid degeneration of superficial to mid-dermal vessel walls that were out of proportion to the inflammation present, particularly neutrophils, and there was no leukocytoclasia; while these changes may be related to the ulceration, they extended into adjacent dermis under intact epidermis. A periodic acid-Schiff stain was negative for fungal elements or vessel wall organisms. Ulceration with equivocal fibrinoid degeneration of vessel walls was noted. The left medial proximal thigh was tested for direct immunofluorescence showing granular deposits of IgM in the walls of the dermal blood vessels.

**Figure 8:**
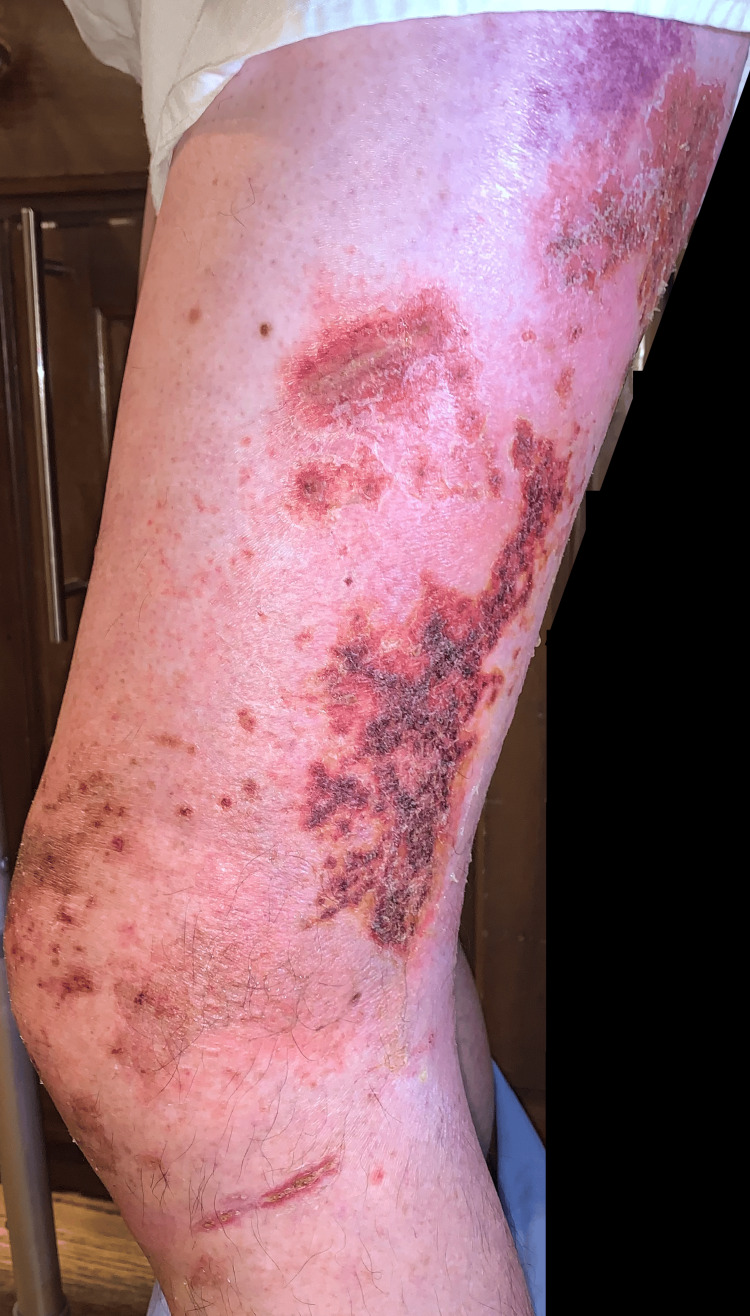
Lateral left leg: additional erythema beyond the plaques, patches, and erosions - developing cellulitic changes in the surrounding tissue

**Figure 9:**
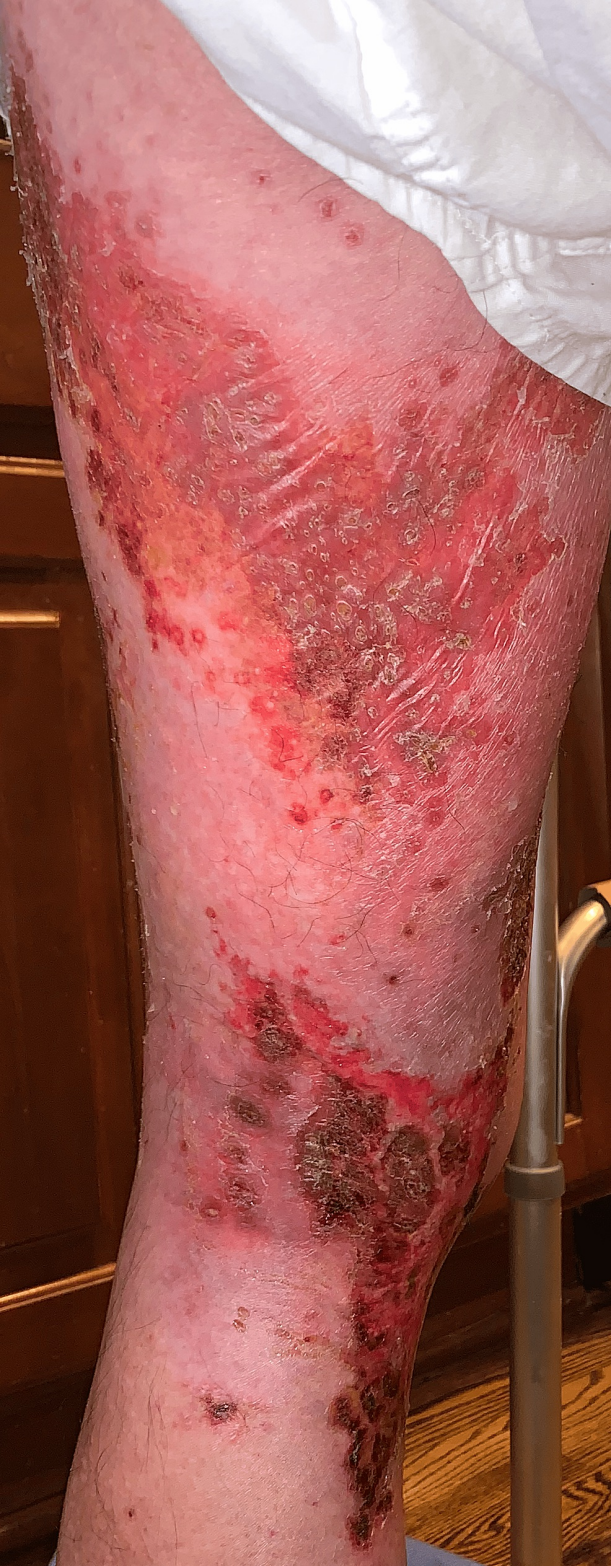
Posterior left leg: erythema beyond the plaques, patches, and erosions - developing cellulitic changes in the surrounding tissue

**Figure 10:**
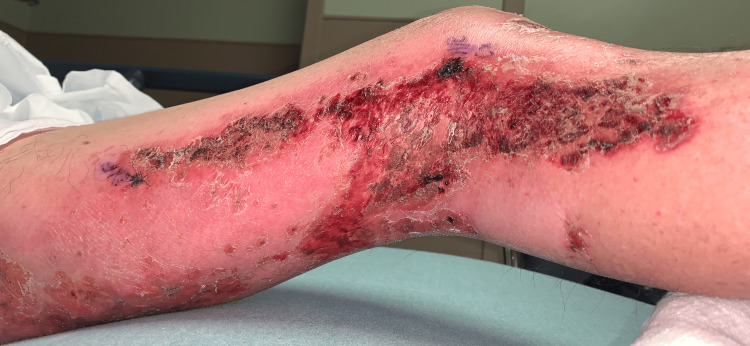
Medial left leg: additional erythema beyond the plaques, patches, and erosions - developing cellulitic changes in the surrounding tissue

**Figure 11:**
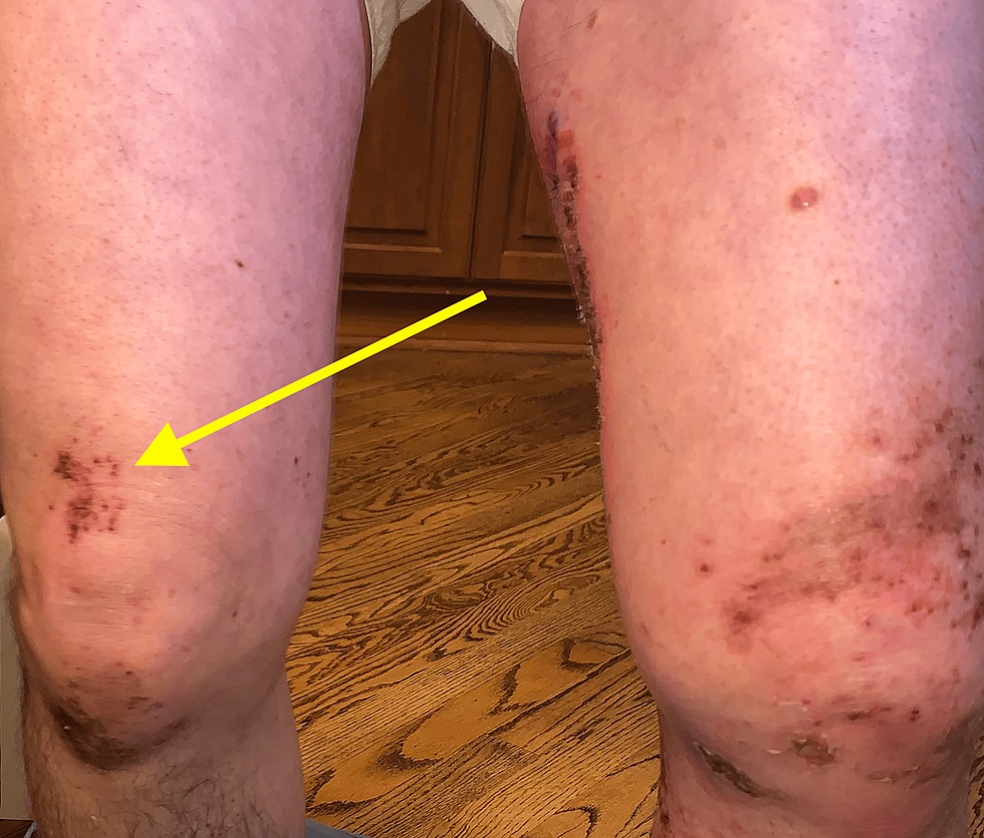
Anterior legs: right superior knee new involvement of purpuric macules outside of L1-L3 dermatome became generalized

**Figure 12:**
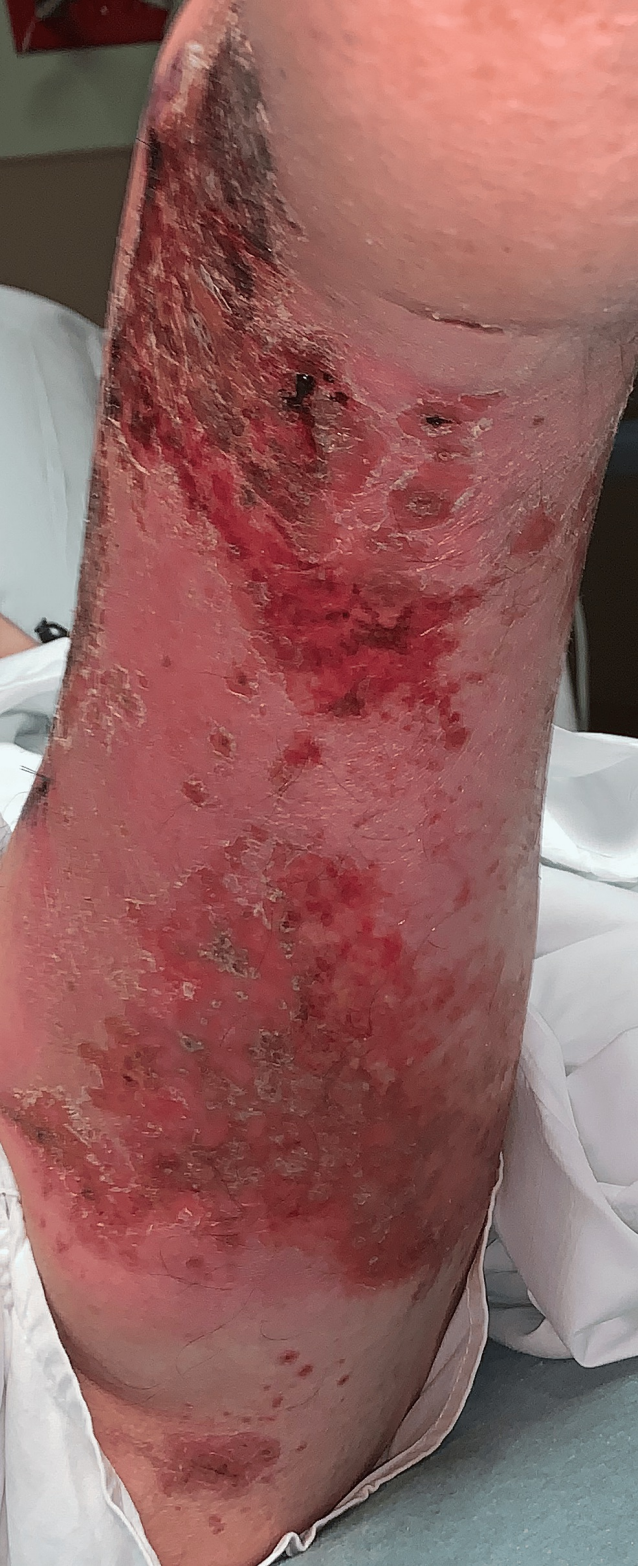
Posterior left leg: additional erythema beyond the plaques, patches, and erosions - developing cellulitic changes in the surrounding tissue

**Figure 13:**
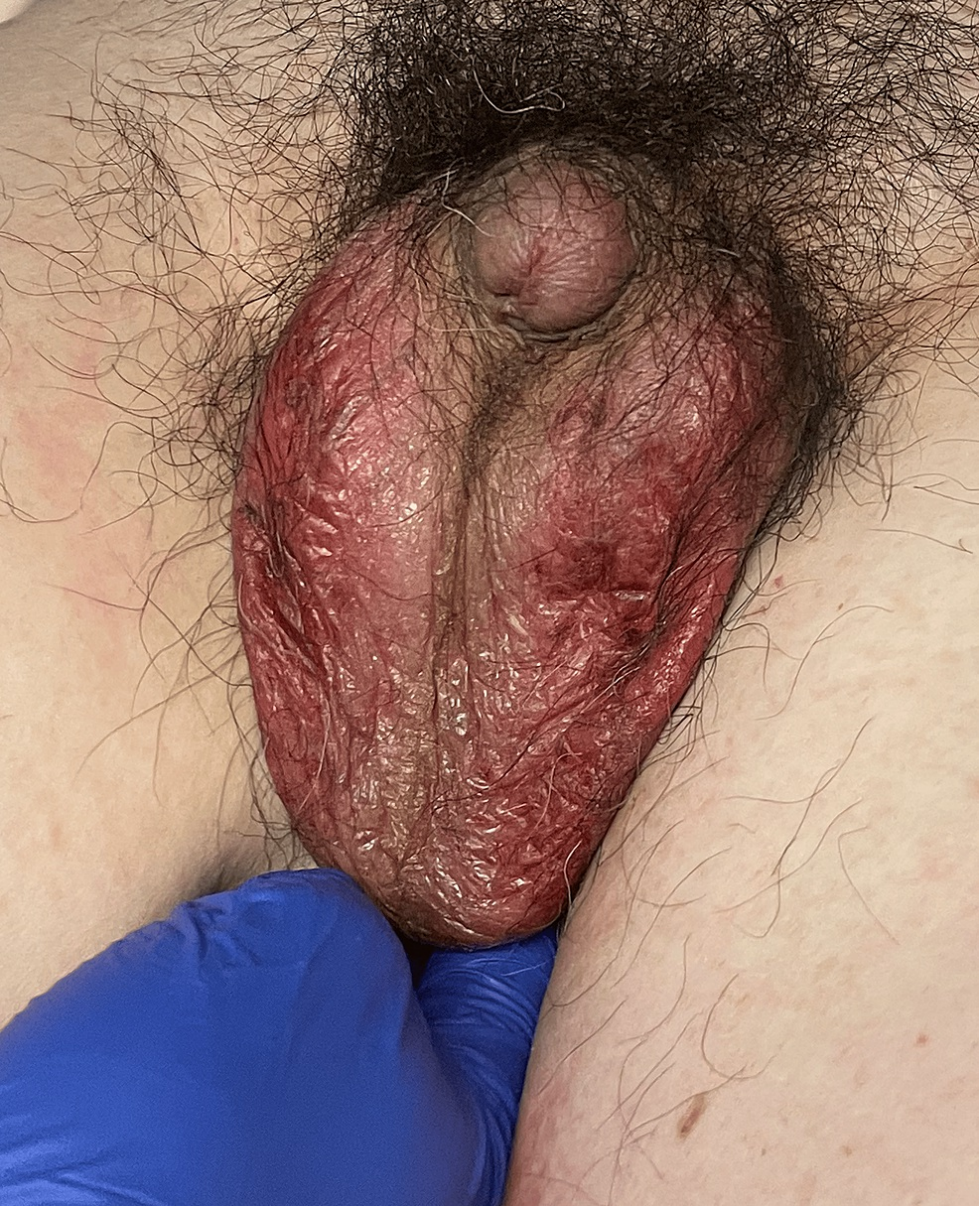
Added bilateral scrotal skin involvement erythema beyond plaques and patches

**Figure 14:**
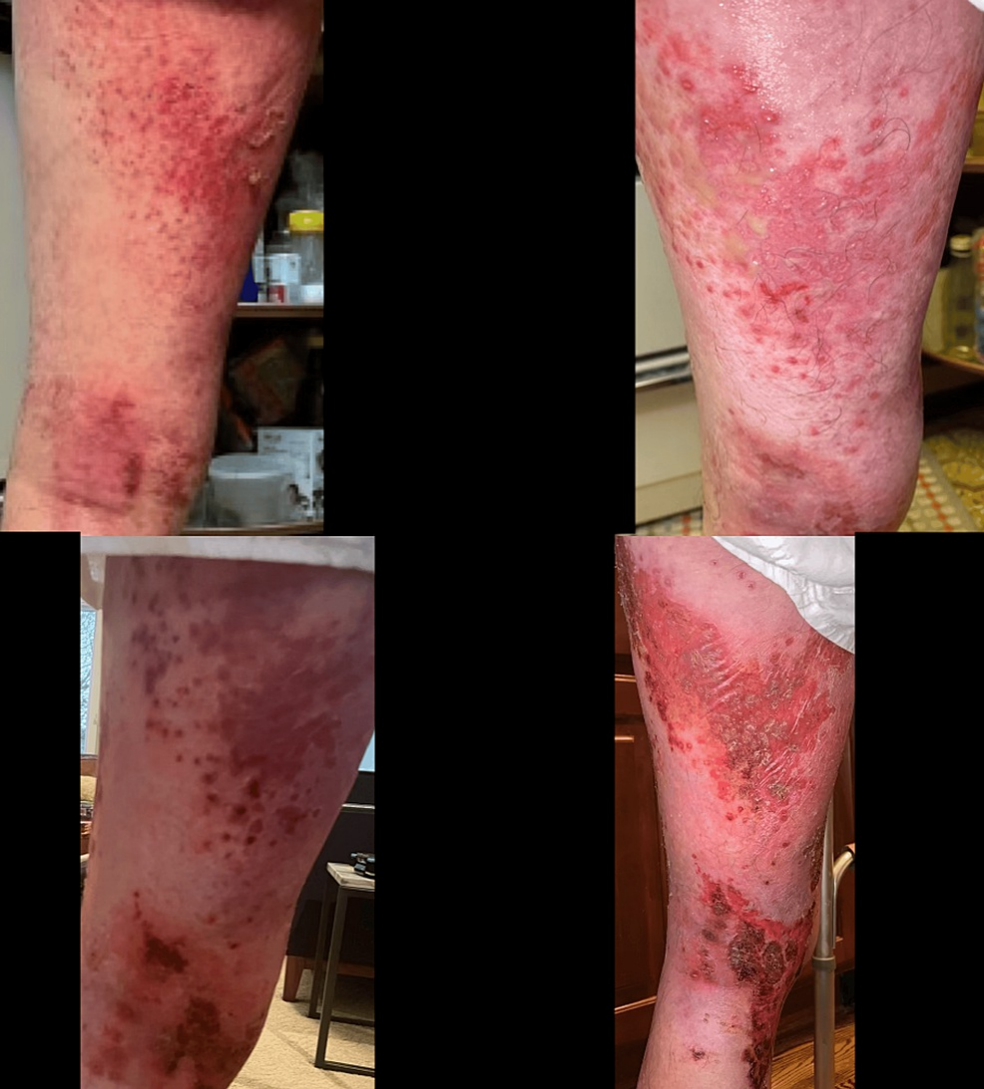
Side-by-side progression of the eruption

**Figure 15:**
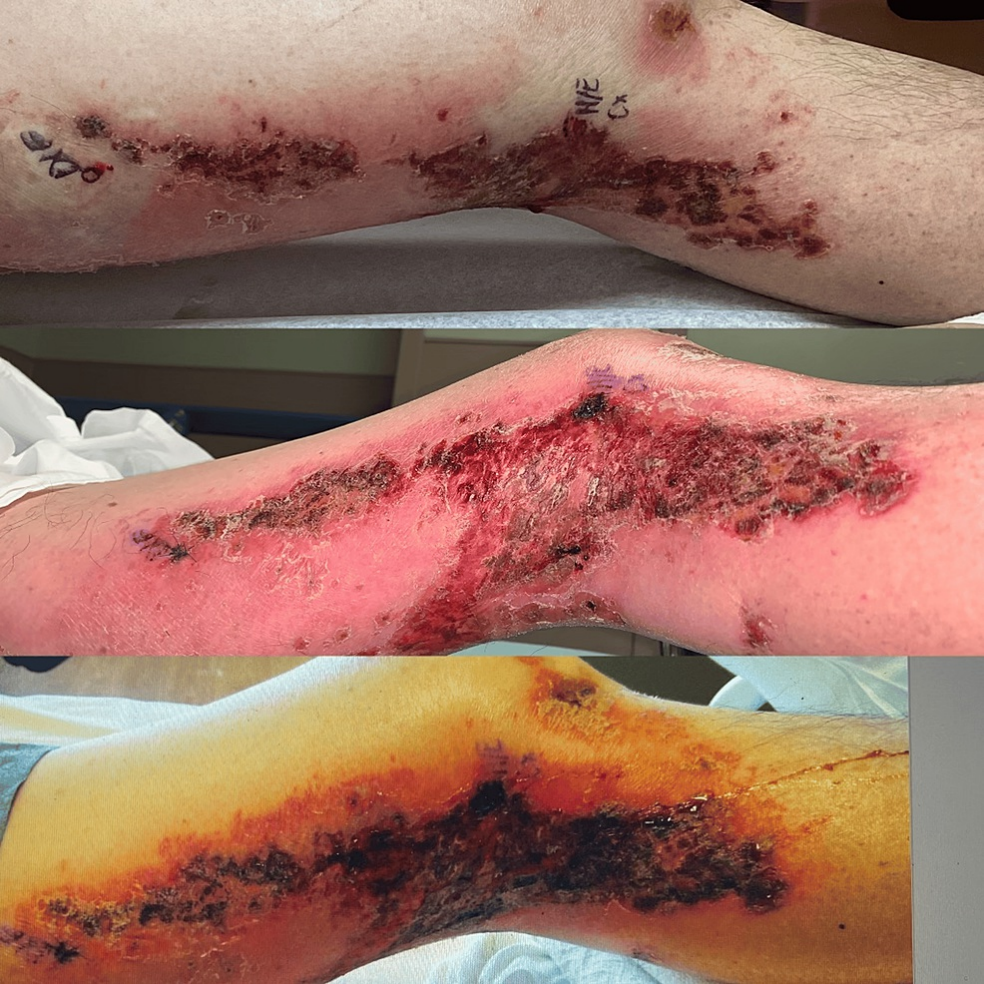
Side-by-side progression of the eruption

**Figure 16:**
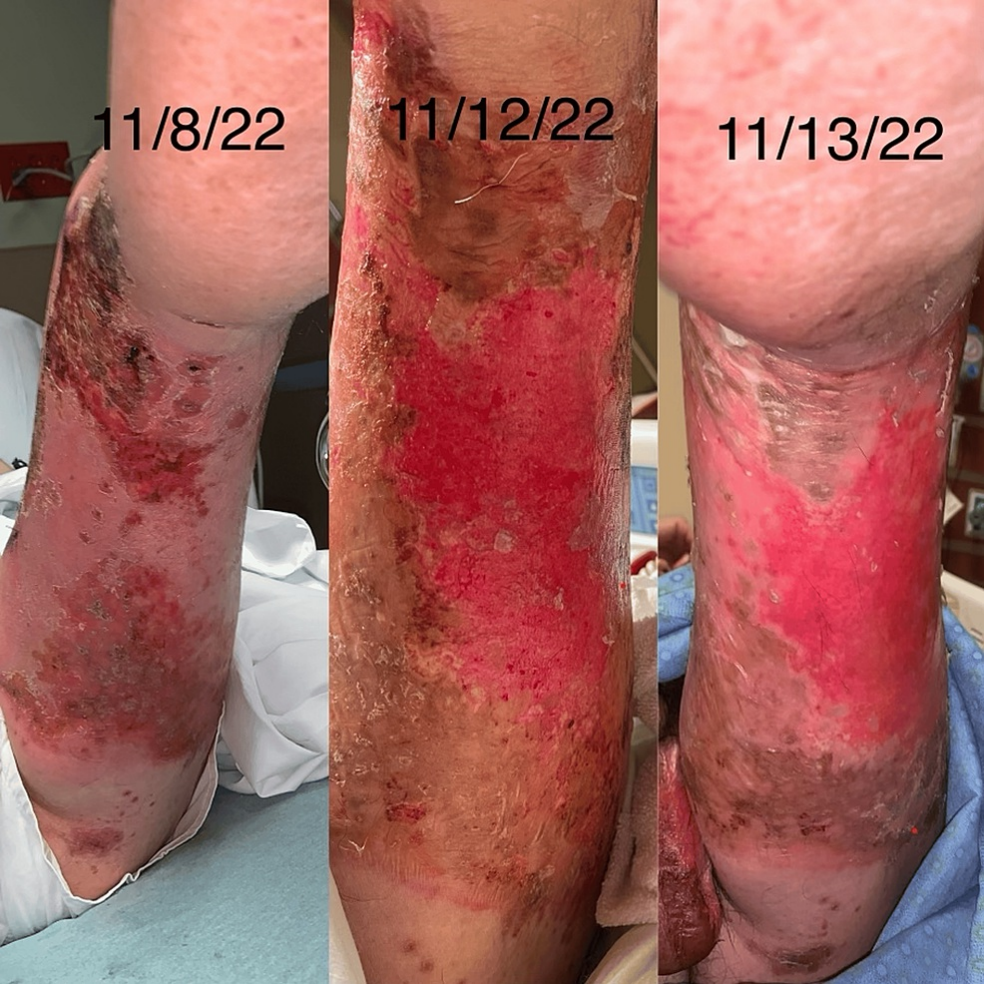
Side-by-side progression of the eruption

**Figure 17:**
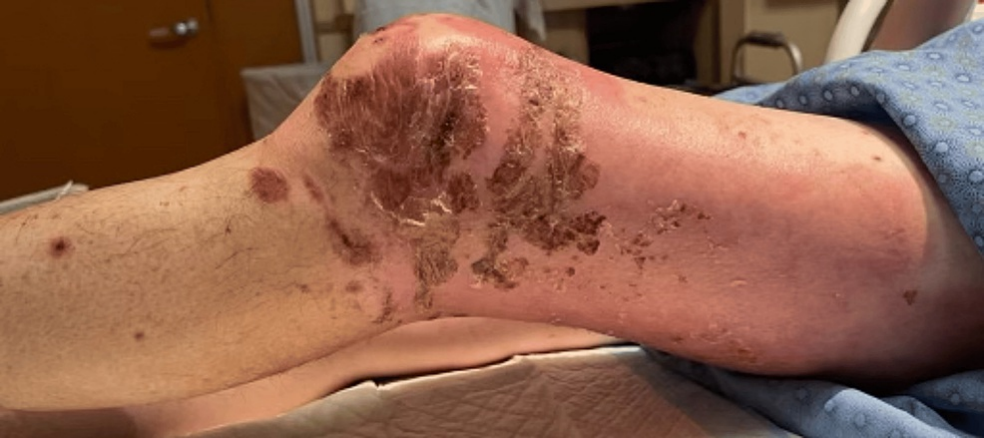
Crusted and healing areas with surrounding post-inflammatory erythema

Two days following the biopsy the patient presented to the ER, due to the development of additional erythema beyond the plaques, patches, and erosions (Figures 8-13). The patient was developing cellulitic changes in the surrounding tissue. Newly developing lesions on the bilateral scrotal skin were involved, as well as added bilateral left lateral malleolus, and right superior knee with the new involvement of purpuric macules outside of L1-L3 dermatome becoming generalized. S1-S2 involvement with punched-out lesions was also noted. With the consideration of possible disseminated herpes zoster with superimposed pseudomonas infection, the patient was admitted to the hospital and started on IV acyclovir, IV vancomycin, and cefazolin. He began improving with treatment. Herpes simplex virus and varicella zoster virus studies came back negative. The culture showed secondary Pseudomonas infection. Lorlatinib and bevacizumab were discontinued to allow for wound healing and prevent further involvement. The progression of eruption is shown side by side (Figures 14-16). Twelve days following his hospitalization the patient continued to improve showing crusted and healing areas with surrounding post-inflammatory erythema (Figure 17). The patient has since achieved 100% improvement on his skin 4-6 months following hospital discharge. He was rechallenged with lorlatinib and stabilized. Later he was rechallenged with bevacizumab and stabilized. He is still on both drugs to date and has remained stable. He has continued to follow up with oncology for NSCLC treatment and management.

## Discussion

Metastatic ALK+ NSCLC can be treated in several ways. In our patient, lorlatinib was used to combat the brain METS and bevacizumab, an anti-VEGF agent, was added to block tumor angiogenesis [1]. There are isolated reports of vascular [1-3] and cutaneous [4] side effects with bevacizumab. There is one case reporting the combination of bevacizumab with FOLFIRI therapy causing a malignant intertrigo reaction [8]. No cases to our knowledge document TEC reactions with bevacizumab alone. The combination of lorlatinib and bevacizumab is relatively new and has limited available data regarding combined adverse effects [7]. In our patient, he began initially experiencing a mild eczematous process that escalated after the initiation of bevacizumab. His eczematous reaction transformed into what appeared to be a vasculitic type reaction with superimposed infections exacerbating it. The distribution and characteristics suggest a vasculitic TEC process, with mixed malignant intertrigo characteristics. The patient’s punch biopsies confirmed a vasculitic-like process. His reaction appeared to have a secondary superimposed disseminated herpes zoster infection, which improved with acyclovir and valaciclovir treatment, despite a negative herpes culture. He also had a culture-positive superimposed pseudomonas infection, which was successfully treated. It is difficult to determine if it was the addition of bevacizumab to lorlatinib alone that initiated the reaction, or if the superimposed infections played a role in the transformation. To our knowledge lorlatinib does not have TEC and/or vasculitis-like reactions associated with it, leaving bevacizumab and the superimposed infection to be the likely culprit. Both medications were discontinued initially. Ultimately, the patient was rechallenged with lorlatinib without significant side effects or complications. He later was rechallenged with bevacizumab and has remained stable, leaving the potential for the trifecta of lorlatinib with bevacizumab combined with superimposed infections to be responsible for the reaction. To our knowledge, this type of reaction has not been reported in the literature related to this therapy.

## Conclusions

Combined anti-cancer therapy has the risk of off-target effects and drug reactions. In cancer patients, the risk for superimposed infections and other complications is increased. Today we report a vasculitic TEC transformation following the initiation of bevacizumab to lorlatinib in a patient with stage 4 ALK+ NSCLC. We hope that this report can add to the body of knowledge surrounding drug-drug interactions, TEC, and drug-induced vasculitic reactions.
